# Shared bias in H chain V-J pairing in naive and memory B cells

**DOI:** 10.3389/fimmu.2023.1166116

**Published:** 2023-09-18

**Authors:** Reut Levi, Shirit Dvorkin, Yoram Louzoun

**Affiliations:** Department of Mathematics, Bar Ilan University, Ramat Gan, Israel

**Keywords:** B cells, rearragement, V genes, selection, J genes, structural

## Abstract

**Introduction:**

H chain rearrangement in B cells is a two-step process where first *D_H_* binds *J_H_*, and only then *V_H_* is joined to the complex. As such, there is no direct rearrangement between *V_H_* and *J_H_*.

**Results:**

Nevertheless, we here show that the *V_H_* JH combinations frequency in humans deviates from the one expected based on each gene usage frequency. This bias is observed mainly in functional rearrangements, and much less in out-of-frame rearrangements. The bias cannot be explained by preferred binding for *D_H_* genes or a preferred reading frame. Preferred *V_H_ J_H_* combinations are shared between donors.

**Discussion:**

These results suggest a common structural mechanism for these biases. Through development, thepreferred *V_H_ J_H_* combinations evolve during peripheral selection to become stronger, but less shared. We propose that peripheral Heavy chain *V_H_ J_H_* usage is initially shaped by a structural selection before the naive B cellstate, followed by pathogen-induced selection for host specific *V_H_*-*J_H_* pairs.

## 1 Introduction

The humoral adaptive immune system is composed of B cell clones carrying different B cell receptors (BCR) (1). Within each clone, specific B cells can further differ through somatic hypermutations (SHM) (2) and affinity maturation. These diverse BCR are often denoted the B cell repertoire and the sequencing of such repertoire is often denoted rep-seq (3). A wider repertoire is argued to be helpful to recognize more antigens, and as such protect the body more efficiently against pathogens (4). Thus, the variety and diversity of this repertoire may be crucial for host health (5). Each BCR is composed of heavy (H) and light (L) chains, with the H representing most of the diversity (6). This diversity is obtained initially by the combination of *V_H_* (variable), *D_H_* (diversity), and *J_H_* (joining) gene segments out of many candidates available. The diversity ofthe L chain is the result of only V-J pairing and junctional diversity (7–9).

The diversity of the H chain is also affected by the choice among multiple possible *D_H_* genes. In contrast with T cells, *D_H_* genes have a large germline diversity contributing to the total repertoire diversity (10), together with the junctional diversity in the *V_H_*-*D_H_* and *D_H_-J_H_* junctions. This initial repertoire is modified through multiple selection stages either within the bone marrow or in the peripheral blood and lymph nodes to produce the observed mature naive, memory, and plasmablasts repertoires (11).

The Pre- and Pro-B cell repertoires resulting from V(D)J rearrangement are far from being random, and they differ from both the out-of-frame (OF) and naive repertoires, which in turn differ from the memory repertoire (12). The difference between naive and memory repertoires may be the result of peripheral selection mechanisms, including affinity maturation and SHM (13). However, the mechanisms shaping the repertoire between the pro and pre-B cells and the naive B cells are still not well characterized.

We here focus on a specific aspect of the repertoire – the pairing between *V_H_* and *J_H_* gene usage in the H chain. Restricted V(D)J usages are associated with different important diseases. Among many others, South Indian patients with precursor B-cell acute lymphoblastic leukemia frequently use specific *V_H_*-*D_H_*-*J_H_* rearrangements (14). *V_H_* 1-69 and *V_H_* 4-59 genes are over-expressed in hepatitis C virus (HCV) related B cell disorders and there is a disease-associated *V_H_D_H_J_H_* usage in HCV patients without clinically detectable lymphoproliferation (15). B lymphocytes (PBL) cells of HIV-infected patients indicate a decrease of the VH3 gene subfamily expression (16).

We have previously shown such a strong bias in *V_B_* and *J_B_* pairing in T cells (17), based on structural selection in the *β* chain. We hypothesize a similar bias in B cells towards specific *V_H_-J_H_* combinations that are more frequent than expected from the *V_H_* and *J_H_* probabilities. Such a pairing has been previously described in the L chain. The L chain lacks a D segment, it can go through multiple V-J rearrangements producing an expected pairing between V and J (18, 19). However, the BCR heavy chain is composed of multiple ordered *V_H_*, *D_H_* and *J_H_* gene segments. The first recombination event in the heavy chain is *D_H_-J_H_* recombination, followed by the joining of *V_H_* segment. Therefore, there is no direct link between the *V_H_* and *J_H_* segments (20). The rearrangement of *D_H_* and *J_H_* may depend on their rearrangement signal, since different heptamer or nonamer combinations may have distinct rearrangement probabilities. The same holds true for *D_H_* and *V_H_*. However, in the H chain, there is no direct rearrangement between *V_H_* and *J_H_*. As such, unless mediated by the *D_H_*, one would not expect *V_H_* to have a preferential bias to specific *J_H_* genes. Moreover, following the rearrangement, the entire *D_H_* locus is erased (except for the rearranged *D_H_* gene). As such, in contrast with the L chain, there is a single rearrangement step, and not consecutive rearrangements that may induce a preference for distal to distal *V_H_* to *J_H_* binding (19, 21). Furthermore, following the deletion of the *D_H_* genes during rearrangement, a single rearrangement step can occur. Thus, one could expect *V_H_* and *J_H_* usage to be independent in the H chain. We show here that this is not the case. Instead, specific *V_H_* genes are consistently associated in different donors with the different *J_H_* genes.

**Figure 1:**
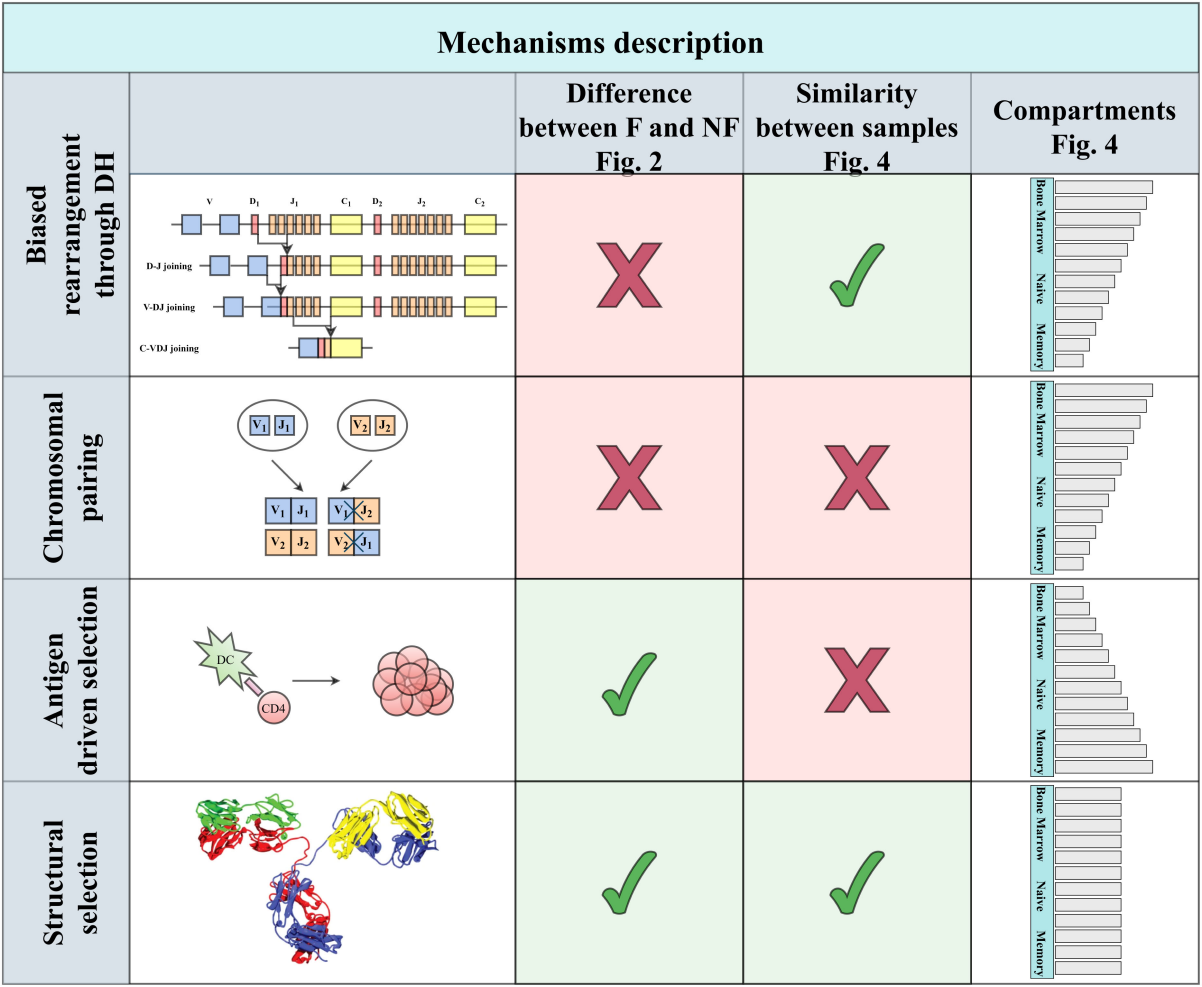
Four main types of explanations can be proposed. The first two are genetic: Either some bias in rearrangement or difference in haplotypes in the two chromosomes, leading to biases following the pairing only within a chromosome. These two mechanisms are expected to affect functional and non-functional clones similarly. While the first is expected to lead to similar biases among patients, the second is expected to differ, based on the chromosomal composition. An alternative mechanism may be antigen-driven selection that will be limited to functional rearrangement, and mainly in the memory compartment, in contrast with the first two that will be mainly in the naive repertoire. Finally, structural selection on the properties of the resulting H chain will lead to similar patterns among patients that will be mainly in the functional compartment, as indeed occurs.

A simple pairing mechanism between *V_H_* and *J_H_* could emerge from the *D_H_* germline diversity. Assume that a specific *J_H_* binds preferentially a specific *D_H_* and similarly a specific *V_H_* would bind preferentially the same *D_H_*, the *V_H_* and *J_H_* would then appear to preferentially pair one with the other. We here show that such a mechanism does not explain the strong pairing among functional rearrangement. Similarly, antigen-induced selection cannot explain the observed bias in early developmental stages. We suggest that as in the case of T cells, this selection is induced by a preference for specific structures, and show an association between the length and polarity of *V_H_* - *J_H_* pairs and their selection (**Figure 1**).

## 2 Methods

### 2.1 Notation

We used the following notation throughout the analysis.

**Table:** 

*V_H_*	V gene in BCR
*J_H_*	J gene in BCR
*P*(*V_H_*)	The probability that a *V_H_* gene appears in a sample
*P*(*J_H_*)	The probability that a *J_H_* gene appears in a sample
*P*(*V_H_,J_H_*)	The probability that a (*V,J*) pair appears in a sample
M(V_H_,J_H_)	*P*(*V_H_, J_H_*) - *P*(*V_H_*) *P(J_H_*)
*C*(*i,j*)	Correlation between *M_i_* (*V_H_,J_H_*) and *Mj*(*V_H_,J_H_*) of samples *i* and *j* over all gene combinations

### 2 Samples studied

We used one published sample Peripheral Repertoire (PREP) (22) and one partially published sample Human Pancreas Analysis Program (HPAP) (23) from organ donors that do not require an IRB in the US, where the experiment was performed.

#### 2.2.1 HPAP samples

DNA was extracted from cryopreserved single-cell suspensions from HPAP donor (24) spleen samples using a Gentra Puregene kit (Qiagen, catalog no. 158767) following the manufacturer’s instructions. Immunoglobulin heavy chain amplifications were performed on gDNA using primers situated in FR1 and JH as described previously (25, 26) Sequencing was performed using an Illumina 2× 300-bp paired-end kit (Illumina MiSeq Reagent Kit v3, 600-cycle, Illumina MS-102-3003). Additional data on HPAP samples can be found on PANC-DB (https://hpap.pmacs.upenn.edu).

Reads were filtered, annotated, and grouped into clones according to the AIRR protocol (see full AIRR Protocol (26). Briefly, paired-end reads were aligned using pRESTO v0.6.0 (27). Short and low-quality reads were removed, and low-quality bases were masked (a quality score threshold of 20). IgBLAST v1.17.0 was used to align and annotate the resulting high-quality sequences (28), using the IMGT (Jan 2019) as a reference (29). ImmuneDB v0.29.10 (30) was used for clonal inference and downstream analysis. Clones were defined as sequences with similar *V_H_* gene, *J_H_* gene, and CDR3 length from each donor that were clustered using hierarchical clustering and had85% or higher similarity in their CDR3 amino-acid sequence. Clones with one sequence copy at the subject level were removed.

#### 2.2.2 PREP

Following Rubelt et al. (22), we analyzed the data in (31). Briefly, participants signed an informed consent under ethical approval (KEK-ZH 2015-0555 and EKNZ 2015-187). Blood samples (5-9 mL) were collected from 53 healthy participants at a single time point. The patients were aged 6 months to 50 years. Sequencing and preprocessing of the data were performed as in (31). In short-RNA was amplified using VH FR1 and P5 primers, and sequences on an Illumina platform. All details are available in (31).

### 2.3 Association measure between V_H_ and J_H_

For each sample, the observed relative frequency of all (*V_H_,J_H_*) pairs *P *(*V_H_,J_H_*) and the expected frequency assuming random pairing were compared. The latter was calculated as the product of the relative frequencies of *V_H_* and *J_H_*, *P*(*V_H_*)*P*(*J_H_*). We computed for each sample:


(1) $$M\left(V_{H},J_{H}\right)=P\left(V_{H},J_{H}\right)-P\left(V_{H}\right)P\left(J_{H}\right).$$

The probabilities were defined per sample and at the clone level (i.e., using only clones in this sample, where *P*(*V_H_*) is defined to be the number of clones with this specific *V_H_* divided by the total number of clones in the sample); and irrespective of the clone size, each clone was counted once. In our analysis of the *M*(*V_H_,J_H_*) distribution, we converted all the values to percentages by multiplying them by 100. Only *V_H_* and *J_H_* genes appearing in the sample were considered (i.e., if a gene was completely absent from a sample, it was ignored).

For example, assume a sample with 10 clones, 2 genes of *V_H_* (V1, V2), and 2 genes of *J_H_* (J1, J2), where we have 6 clones with V1, 4 clones with V2, 7 clones with J1 and 3 clones with J2. At the pair level, there are 4 (V1, J1), 2 (V1, J2), 3 (V2, J1)and one (V2, J2) clones. In order to calculate *M*(*V*1,*J*2), we first calculate *P*(*V*1,*J*2), *P*(*V*1) and *P*(*V*2). In our case, *P*(*V*1,*J*2) = 0.2 - 2 clones divided by the total of 10 clones in the sample. Similarly, *P*(*V*1) = 0.6 and *P*(*J*2) = 0.3. Therefore, M(V1,J2)=P(V1,J2)−P(V1)P(J2)=0.2−0.6·0.3=0.02=2% .

### 2.4 Correlation between samples

To measure the similarity in the deviation from a random pairing between different samples, we calculated the Spearman correlation coefficient for all possible pairs of samples based on the *M*(*V_H_,J_H_*) values.

Given two samples, *i* and *j*, where each contains a subset of the *V_H_* and *J_H_* genes *V_H_i_k_, J_H_i_K_*, V_H_j_k_, J_H_j_k_. For each pair of samples, the common (V_H_,J_H_) pairs were taken s.t.


(2) $$S=\{\left(V_{H},J_{H}\right)|V_{H}\in V_{H}i_{k}\wedge V_{H}\in V_{H}j_{k}\wedge J_{H}\in J_{H}i_{k}\wedge J_{H}\in J_{H}j_{k}\}.$$

We first computed the *M_i_* (*V_H_,J_H_*) and *M_j_* (*V_H_,J_H_*) for all pairs in the set *S*. Then, we calculated the Spearman correlation for these pairs:


(3) $$C\left(i,j\right)=\rho_{\text{Spearman}}\left(M_{i}\left(V_{H},J_{H}\right),M_{j}\left(V_{H},J_{H}\right)\right).$$

For example, suppose we have 2 samples (*i.j*) with two genes of *V_H_* and *J_H_* each (V1, V2, J1, J2), where all the pairs (V1, J1), (V1, J2), (V2, J1), (V2, J2) exist in both files. We further assume that we obtained that *M*(*V_H_,J_H_*) is 0.2, 0.4, 0.1,0.6 for the pairs above in the first sample and 0.1, 0.5, 0.2, 0.8 in the second sample. We determine how similar the deviation from random pairing is between the two samples by calculating the correlation between their respective vectors. i.e.,…

### 2.5 Detection of specific V_H_-J_H_ pairs that deviate from random pairing

We calculated the probabilities *P*(*V_H_,J_H_*) and *P*(*V_H_*)*P*(*J_H_*) for each (*V_H_,J_H_*) pair across all samples to identify any specific pairs that deviated from the null model of random pairing. Next, over all samples, a paired T-test on *P*(*V_H_,J_H_*) and *P*(*V_H_*)*P*(*J_H_*) was performed for each pair separately. Finally, we used the Benjamini-Hochberg correction benjamini1995controlling to adjust the obtained *p*-values. We considered pairs with a corrected *p*-value less than 0.01 as significant.

### 2.6 Null models

To generate a null model for our analysis, we scrambled the *V_H_* and *J_H_* segments within the *V_H_J_H_* pairs. This involved randomly reassigning the *V_H_* genes of the different clones in each sample. Specifically, we listed all clones and performed a permutation on the *J_H_* gene associated with each *V_H_* gene within a given sample. Scrambling was performed at the clone level, and not at the read level (i.e. we did not scramble reads within a clone). We ignored the clone size in current the analysis.

### 2.7 Biochemical features

We used only the functional (F) clones for the HPAP dataset, and each isotype separately (IGHA, IGHD, IGHG and IGHM) for the PREP dataset. For each possible pair in a given file, we calculated the combined lengths of *V_H_* and *J_H_*. Furthermore, we calculated the total Kyte Doolittle (KD), Molecular Weight (MW), and Isoelectric Point (IP) values for every CDR3 amino acid in each pair and averaged the results. This was done for each file and pair. Next, we determined the *M*(*V_H_,J_H_*) values for each *V_H_,J_H_* pair within a given file and computed the average for all pairs in the dataset. A Spearman correlation coefficient was then computed between the mean *M*(*V_H_,J_H_*) and the sum of all gene lengths, KD, MW, and IP values.

### 2.8 Statistical analysis

- In order to evaluate the correlation between different samples, only the pairs that were present in both samples were considered. For each pair (*V_H_,J_H_*), we computed both *M*(*V_H_,J_H_*) and *M*_1_(*V_H_,J_H_*). Here, *M*_1_ is the metric used for themixed data in both the real data and the null model. Next, we computed the *Spearman correlation coefficient* for these two sets of data.
- The *two-sided Kolmogorov-Smirnov statistic on two samples* (32) was used to test whether the distribution of *M*(*V_H_,J_H_*) on the real data differs from that in the null model.
- To examine whether the standard deviation of *M*(*V_H_,J_H_*) on the real data is different from the standard deviation of *M*(*V_H_,J_H_*) on the null model, we used a *two-sided T-test on two related samples of scores*. Using this test, we werealso able to determine which pairs have a signal. *P*(*V_H_,J_H_*) and *P*(*V_H_*)*P*(*J_H_*) were calculated and the above test was performed separately for each pair (*V_H_,J_H_*) and *P*(*V_H_*)*P*(*J_H_*). We applied the *Benjamini-Hochberg correction* for multipletests (33).
- To evaluate whether the distribution of correlations in the functional (F) clones, non-functional (NF) clones, and null model significantly differed, we performed a *one-way* ANOVA test on the correlation values. In addition, to test whether the correlations within a patient are distinct from the correlations across different patients, we applied the *two-sided T-test for the means of two independent samples of scores*.
- We used a *one-way Chi-square test* in order to check whether the (*V_H_*,*J_H_*) pairs with the most significant deviation from random pairing (*p*<0.01) over and under-represented are consistent across datasets.
- To test the correlations between *M*(*V_H_,J_H_*) and the biochemical features, we divided the data into 20 bins based on the chemical values distribution (KD, MW, IP, and sum of genes lengths) and used a *Wilcoxon signed-rank test*, for *M*(*V_H_,J_H_*) for each bin.

### 2.9 Mutual Information

The Mutual Information between two random variables *X* and *Y*, denoted as *MI*(*X;Y*), is defined as the reduction in uncertainty about one variable (e.g., *X*) given the knowledge of the other variable (e.g.,*Y*). In other words, it measures how much knowing the value of one variable helps us in predicting the value of the other variable. It is defined as:


(4) $$MI\left(X;Y\right)=\sum_{y\in Y}\sum_{x\in X}P_{\left(X,Y\right)}\left(x,y\right)log\left(\frac{P_{\left(X,Y\right)}\left(x,y\right)}{P_{X}\left(x\right)P_{Y}\left(y\right)}\right),$$

where *p*(*x,y*) is the joint probability function of *X* and *Y*, and *p*(*x*) and *p*(*y*) are the marginal probability mass functions of *X* and *Y*, respectively.

A key property of Mutual Information is that it is non-negative – *MI*(*X*;*Y*) ≥ 0. *MI*(*X*;*Y) = 0* indicates that *X* and *Y* are statistically independent.

- High Mutual Information: *MI*(*X*;*Y)* is close to the maximum value (the minimum between the entropy *x* and of *y*) with a strong dependency between the variables. In this case, knowledge of one variable provides significant information about theother.
- Low or Zero Mutual Information: *MI*(*X*;*Y)* values close to zero indicate that the variables are independent of each other. Knowing the value of one variable does not offer any useful information about the other.

## 3 Results

### 3.1 V_H_,J_H_ usage is biased in the H chain

**Figure 2:**
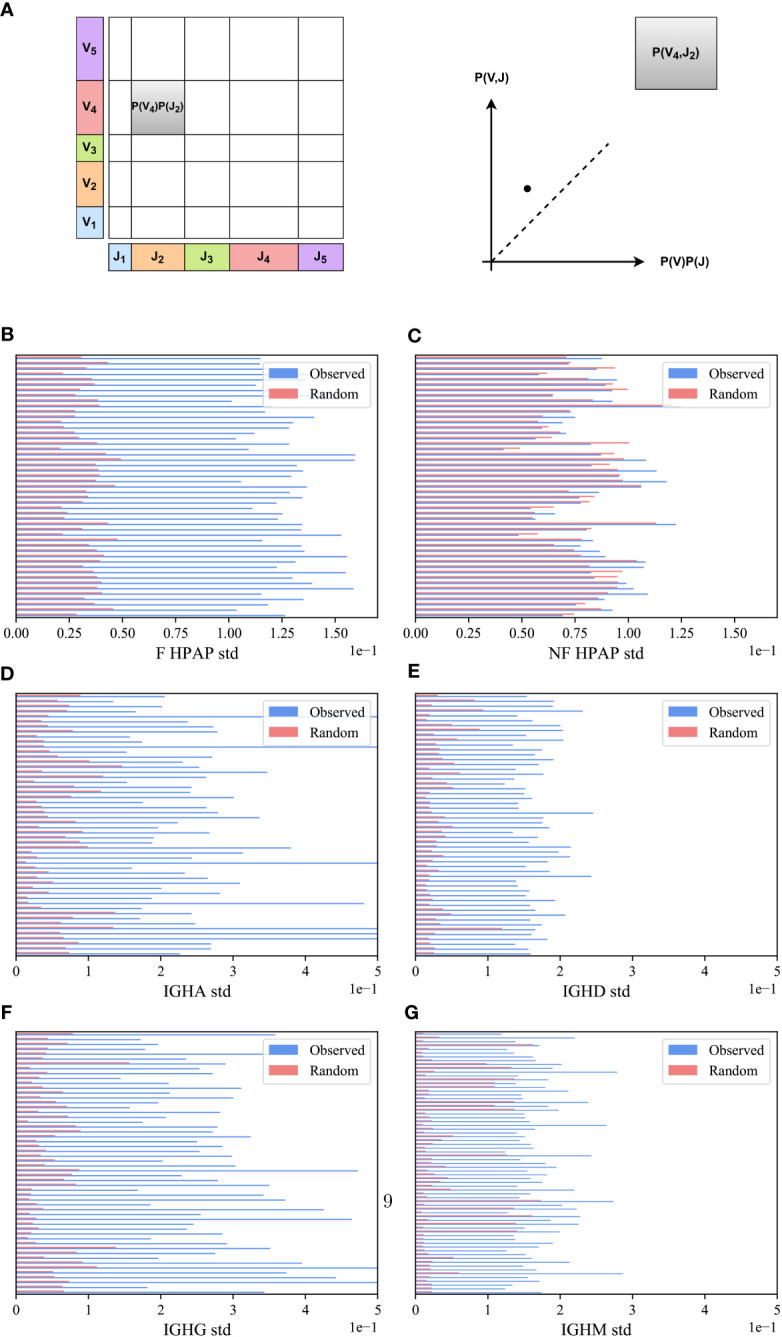
*M(V_H_,J_H_)* bias. **(A)** Schematic explanation *M*(*V_H_,J_H_*) measure. We calculated the proportion of clones using a particular *J_H_* and *V_H_* gene in a sample and represented it as a point on a graph, where the *J_H_* gene proportion is on the x-axis and the *V_H_* gene proportion is on the y-axis. We multiplied these proportions (i.e., the size of rectangles) and compared them with the actual number of clones that used a specific *V_H_,J_H_* gene pair. **(B–G)** The standard deviation (std) of *V_H_,J_H_* values for the HPAP dataset **(B, C)** and the PREP dataset for each isotype separately **(D–G)**. The blue bars describe the real F clone values **(B, D–G)** and the real NF clone values **(C)** while the pink bars represent the null model. Each row represents a sample in the HPAP dataset.

To study whether *V_H_-J_H_* pairing deviates from the random pairing, we used 2 sample sets, denoted HPAP and PREP datasets. Each dataset contains several patients (see Methods). The HPAP sample contains functional and non-functional clones. The PREP dataset contains only functional clones. The dataset underwent a filtering step, where all samples with less than 1,000 clones were removed. To avoid biases introduced by differential amplification, the frequency of each clone in each donor was ignored during the analysis (the results at the single sequence level, and not at the clone level are similar and presented in **Supplementary Material Figure S1**). Using two fields gene notation (e.g., V01-02 and J01-02), *V_H_* gene and *J_H_* gene representations were grouped, and allelic differences were ignored (V01-02:01 → V01-02). We will further show that the deviation from random pairing is not the effect of allele differences (**Figure 2**). The second dataset is a set of peripheral repertoire divided into memory and naive clones and further divided by isotypes, denoted here as PREP.

We compared the *V_H_,J_H_* frequency distribution of functional (F) clones in each sample in the HPAP dataset and for each isotype separately (IGHA, IGHD, IGHG and IGHM) in the PREP dataset with the one expected under the null hypothesis of independent pairing. Specifically, we computed the marginal probability of each *J_H_* and *V_H_* gene (i.e., the probability that a randomly chosen clone would have a given *J_H_* - x-axis in **Figure 2A** or *V_H_* gene - y-axis), and multiplied them to obtain the expected value of the pair assuming independence *P*(*V_H_*)*P*(*J_H_*) (shown as the area of the rectangle in **Figure 2A**). As a schematic example, for the pair (*V*_4_,*J*_2_) in **Figure 2A**, the observed *P*(*V*_4_,*J*_2_) is larger (i.e., has more clones) than expected by *P*(*V*4)*P*(*J*_2_) (i.e., it is above the diagonal line in the observed vs. expected plot).

To systematically quantify this deviation, we computed for each (*V_H_,J_H_*) pair in a given sample:


(5) $$M\left(V_{H},J_{H}\right)=P\left(V_{H},J_{H}\right)-P\left(V_{H}\right)P\left(J_{H}\right).$$

*M*(*V_H_,J_H_*) is expected to be zero for random pairing. There can be deviations from zero due to finite-size effects. To test for such deviations, we compared the distribution of *M*(*V_H_,J_H_*) for the real clones to the distribution obtained from the null model, in which the *V_H_* and *J_H_* genes of the clones were randomly reassigned. This random scrambling was performed separately for each sample (see Methods). We observed that the distribution of *M*(*V_H_,J_H_*) for the real clones is consistently wider than the distribution obtained from the null model in all F HPAP and PREP repertoires (**Figures 2B, D–G**).

To quantify the difference between the real and null models of *M*(*V_H_,J_H_*) distributions, we calculated the standard deviation (std) and applied a paired T-test on the standard deviations of the real and null models across all samples in each dataset. The standard deviation of the functional (F) clones is consistently larger than the null model in all samples (**Figures 2B, D–G** for the HPAP and the PREP datasets, *p*-value 1*e*-10 for both datasets). To confirm the significant difference, a Kolmogorov-Smirnov test (32) on the distributions in the real data and the null model for all samples together was performed, which also yielded a very significant difference (p-value<1*e*-10 for both datasets).

In order to validate that the bias of *V_H_,J_H_* usage is not a result of genetic factors of different alleles on different chromosomes, we tested whether the bias in usage also exists in the non-functional (NF) clones. We computed the *M*(*V_H_,J_H_*) in the non-functional HPAP dataset, and found that while the distribution of *M*(*V_H_,J_H_*) for the F clones is much wider than for the null model, the results on the NF clones show no such difference. The distribution of *M*(*V_H_,J_H_*) for both the NF clones and for the null model is similar with no significant difference between the standard deviation of the NF clones and the null model (**Figure 2C** for the HPAP dataset, p-value 0.08). Furthermore, there is no significant difference between the distributionsas measured by the Kolmogorov-Smirnov test (*p*-value 0.94). Four simple mechanisms could be argued to explain the bias (**Figure 1**):

- Genetic mechanisms (any mechanism that is purely genetic), such as joint preference for *D_H_* genes by *V_H_* and *J_H_*. Genetic mechanisms would induce such pairing also in NF clones. Also, in such a case, we would expect the conditional distribution of *V_H_, J_H_* given *D_H_* to be independent. We will show that this is not the case.
- Pairing between alleles. Specific *V_H_* and *J_H_* alleles are on one chromosome and have a high expression level. When averaging over both chromosomes, this would look like a bias for them. Again, such a mechanism would affect F and NF clones similarly. In such a case, we would also expect the pairing to differ among individuals. We will further show that this is not the case.
- Antigen-driven selection. We would expect the bias to be limited to memory or activated cells. We further show that such a bias exists and is already large at the naive stage.
- Structural selection for the stability of BCR or basal binding to antigens expressed in the bone marrow.

### 3.2 *V_H_,J_H_* pairing through D_H_

To further disqualify genetic mechanisms, we tested the possibility that the bias is induced by preferred *D_H_* pairing. For example, if a given *D_H_* binds only a given *J_H_* and only a given *V_H_*, then this *V_H_-J_H_* pair will be over-expressed.

To determine if the correlation with *D_H_* genes is indeed the cause of the *V_H_,J_H_* pairing, we compared *P*(*V_H_,J_H_*) vs. two scenarios:

- Random pairing: The probabilities of a *V_H_,J_H_* pair is the product of their marginal probabilities: *P*(*V_H_,J_H_*) ~ *P*(*V_H_*)*P*(*J_H_*).
- Pairing through *D_H_* genes: Suppose that *J_H_* and *D_H_* are paired with certain preferences, and in addition, *D_H_* and *V_H_* are paired with other preferences. One can thus compute: $P\left(V_{H},J_{H}\right)=\sum_{D_{H}}P\left(D_{H},V_{H},J_{H}\right)∼\sum_{D_{H}}P(V_{H}|D_{H})P(J_{H}|D_{H})P\left(D_{H}\right)$ .

**Figure 3:**
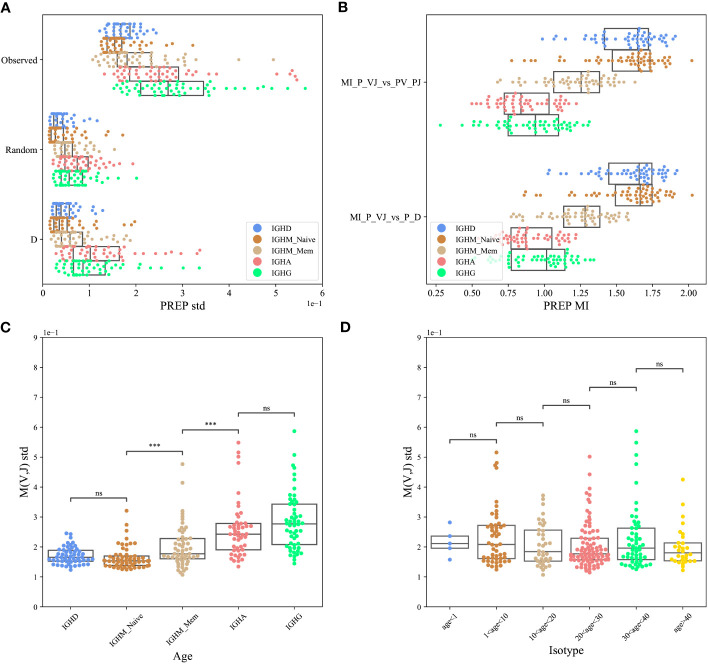
*V_H_*, *J_H_* pairing through D (PREP dataset). **(A)** The standard deviation (std) of the *M*(*V_H_,J_H_*) values for the F clones, where *P*(*V_H_,J_H_*) was either the observed one or computed according to one of the two models described above (random pairing and pairing through *D_H_* genes). The result is shown for each isotype separately. **(B)** The Mutual Information (MI) between the log of the observed and expected *P*(*V_H_,J_H_*) relative frequencies in those three cases for the F clones for each isotype separately. **(C, D)** The standard deviation of *M*(*V_H_,J_H_*) values for eachisotype separately **(C)** and for different age groups **(D)**, where ‘***’: *p*-value<0.001 and ‘ns’: *p*-value ≥0.05.

To test for the effect of D-based pairing, we used the PREP dataset. We computed the standard deviation of $M\left(V_{H},J_{H}\right)=P\left(V_{H},J_{H}\right)-P\left(V_{H}\right)P\left(J_{H}\right)$ , where *P*(*V_H_,J_H_*) was either the observed one, or computed according to one of the two models above, and *P*(*V_H_*)*P*(*J_H_*) is the same for all models. The standard deviation of the observed data is the highest for the real clones (**Figure 3A**), followed by the model based on *D_H_* pairing, followed by the null model (ANOVA test *p*<1*e*-10 for each isotype separately, paired T-test between real clones and the model based on *D_H_* pairing *p*<1*e*-10).

A caveat of the *M*(*V_H_,J_H_*) value is that it is mostly affected by the large clones. To address that, we computed the Mutual Information (MI) between the log of the observed and expected *P*(*V_H_,J_H_*) relative frequencies in the models above for each isotype separately. The real data is similar to the two models above (**Figure 3B**, T-test, *p* > 0.05 for all isotypes - showing that there is no difference between the models)

These results combined with the absence of bias between *V_H_* and *J_H_* in the NF clones suggest that any bias in the F clones is not due to rearrangement or to any purely genetic mechanism.

### 3.3 Development of bias

Any kind of selection affecting the *V_H_* and *J_H_* gene usage is expected to induce deviation from random pairing. As such, even if there is a strong deviation in the naive repertoire, we expect the deviation to grow following antigen-induced peripheral selection. To test that, we compared the standard error of *M*(*V_H_,J_H_*) for isotype-switched cells and for naive and memory IgM repertoires. One can clearly see a consistent development of the bias through development (**Figure 3C**, T-test between following compartments *p*<0.001, between the naive and memory and between the memory and isotype switched cells, but no difference between IGHD and naive IGHM, and no significant difference between IGHG and IGHA). We further tested if the bias is accumulating or decreasing over the age. We found a slight yet non-significant decrease (**Figure 3D**, T-test between following compartment - non-significant).

### 3.4 Correlation of V_H_,J_H_ bias between and within patients and isotypes

**Figure 4:**
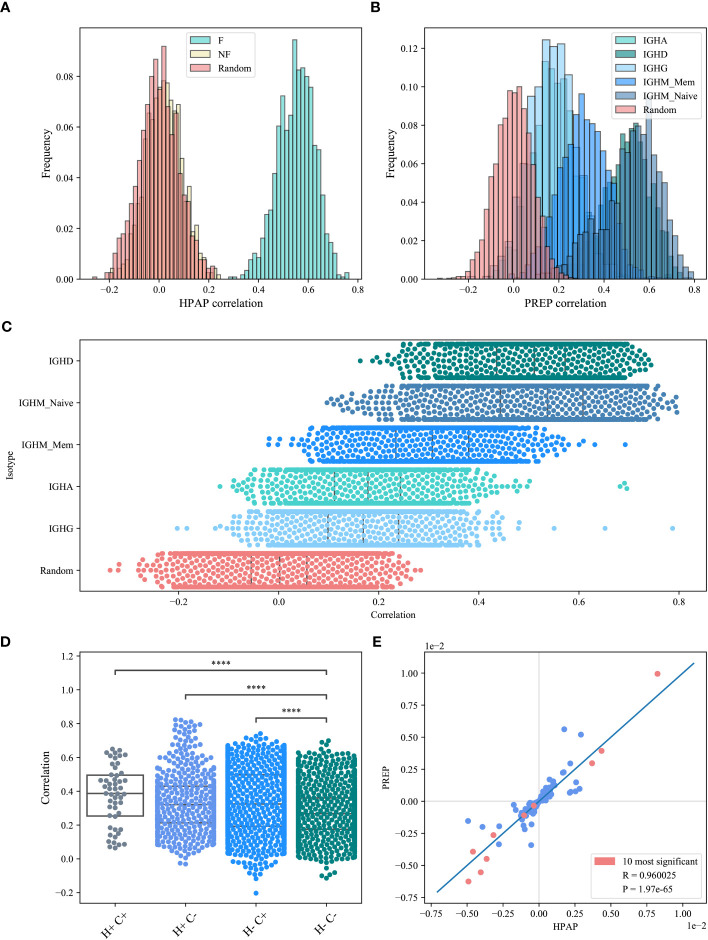
*M*(*V_H_,J_H_*) correlation. **(A, B)** The correlations histogram of the *M*(*V_H_,J_H_*) values for the HPAP dataset **(A)** and the PREP dataset **(B)**. The blue shade histograms represent the F clones, the beige histogram is the NF clones and the pink histogram represents the null model. **(C)** Correlations of *M*(*V_H_, J_H_*) values for the PREP dataset for each isotype separately. **(D)** Correlations of *M*(*V_H_, J_H_*) values for the PREP dataset within and between hosts and compartments, where H+ represents within host, H- represents between hosts, C+ represents within compartment, C- represents between compartments. Star symbols follow the previous plot. **(E)** *M*(*V_H_, J_H_*) values between HPAP and PREP functional datasets. The pink points represent the common pairs of the 10 most significant pairs between these two datasets.

If, indeed, the *V_H_,J_H_* bias is induced by a structural selection, it should be similar across hosts. Alternatively, if the pairing is antigen-driven, or by allele preference, one would expect it to be uncorrelated between hosts. We computed the Spearman correlation coefficient between *M*(*V_H_,J_H_*) values for all sample pairs from different hosts in the HPAP dataset, and computed the distribution of the correlations (**Figure 4A**) for the F clones (blue bars), the NF clones (beige bars) and the null model (pink bars). Only the shared (*V_H_,J_H_*) pairs were taken into account when computing the correlations, for each pair of samples. As expected, the NF and the null model had distribution distributed around 0. In contrast, the correlation histogram of the F clones is centered around 0.5 for the HPAP (ANOVA test *p*<1*e*-10 for all groups).

We repeated the analysis for the PREP samples for each isotype (**Figure 4B**, colored in blue shades). We also split the IGHM isotype into memory and naive and calculated the correlation for each of them separately (**Figure 4C**). The similarity starts high and then decreases through development, suggesting a shared structural selection followed by host-specific antigen-induced selection that increases the deviation from random pairing, but decreases the similarity between repertoires.

We further explored the correlations between the values of *M*(*V_H_,J_H_*) among hosts and isotypes (further denoted as compartment - all isotypes and naive and memory IgM separately) and compared the *M*(*V_H_,J_H_*) correlations within and between compartments and within and between hosts. The highest correlations are indeed within a donor and a compartment (T-test *p*<l*e*-10 vs H-C-), followed by correlations within compartments (H-C+) and the correlations within hosts (H+C-) that were both higher than the one between compartments (H-C-) (0.337 and 0.334 vs 0.27 on average, T-test *p*<le-10 - **Figure 4D**).

If the selection is indeed structural, we expect the over and under-represented pairs to be similar between datasets. We analyzed all the (*V_H_,J_H_*) pairs with the most significant deviation from the null model (*p*<0.01). Indeed, the most significant pairs overlap in the two datasets studied here (117 overlapping pairs vs 63.23 expected randomly, chi-square *p*<1.36*e-*11). In addition, all significant pairs that overlap between the two data sets have the same deviation sign (**Figure 4E**).

We further analyzed the common significant pairs (corrected *p*-value<0.01) between the two datasets, and compared *M*(*V_H_,J_H_*) values among datasets. *M*(*V_H_,J_H_*) is highly consistent among the datasets (Spearman Correlation Coefficient 0.96, *p*<l*e*-10, **Figure 4E**. The pink points represent the common pairs of the 10 most significant pairs between each of the datasets).

### 3.5 V_H_,J_H_ pairing is associated with biochemical properties of receptors

If selection is structural, we expect associations between the *V_H_,J_H_* pairing (as measured by the *M*(*V_H_,J_H_*) values) and the structural properties of the receptors. We computed for the receptor within each (*V_H_,J_H_*) pair in each sample the molecular weight (MW), the average length (defined to be the sum of *V_H_* and *J_H_* genes length in amino acids), the charge (as measured by the iso-electric point - IP), and the hydrophobicity (defined through the kyte doolittle - KD score). The measures were implemented using the contribution of each amino acid (AA) to the score, as defined by the Biopython package (34). We analyzed the full CDR3 sequence, and not only the *V_H_*, *J_H_* or *D_H_* genes, since those are not clearly defined, and the *D_H_* gene is often ambiguous.

**Figure 5:**
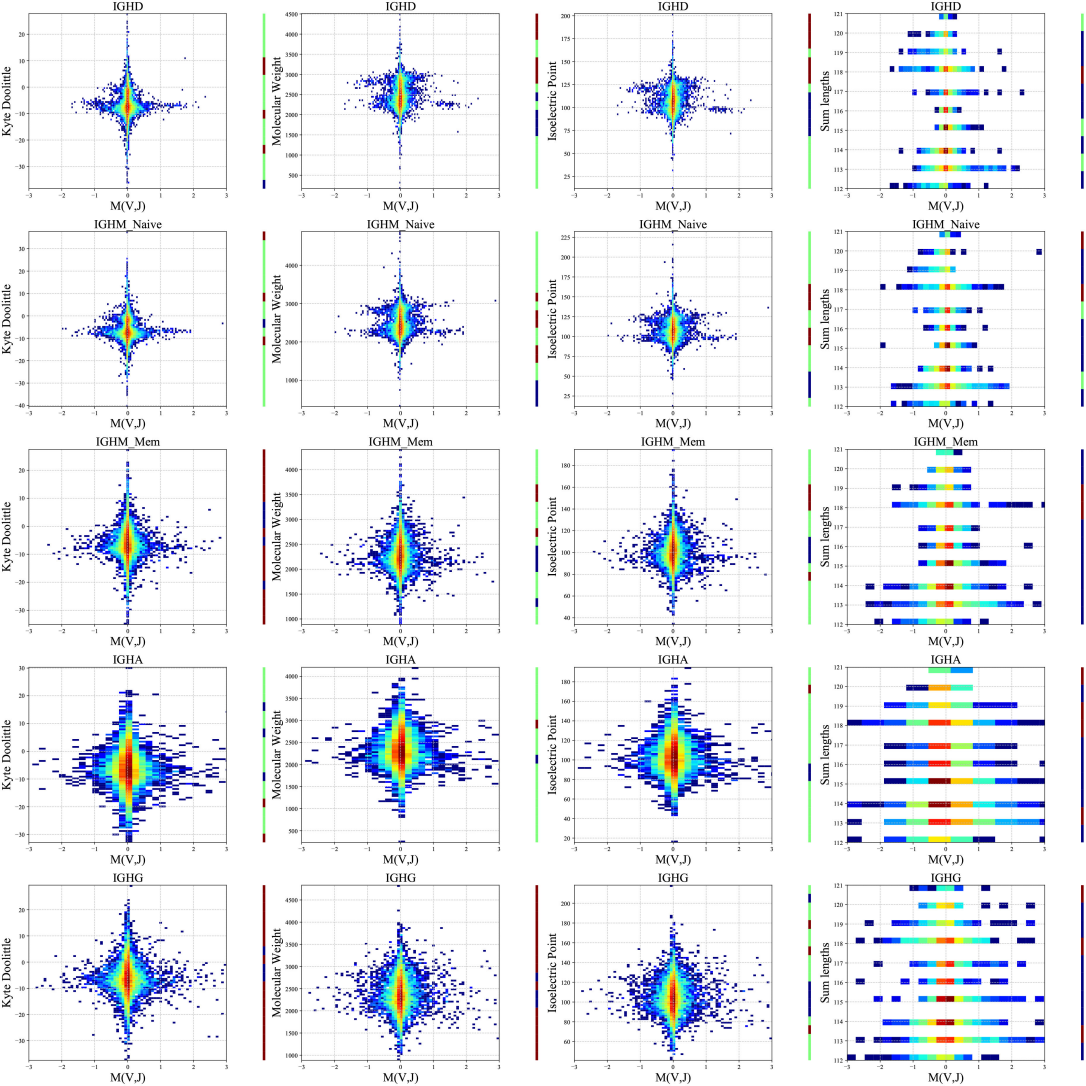
Two-dimensional histogram (PREP dataset). 2D histogram where the x-axis represents the *M*(*V_H_,J_H_*) values for each group separately (isotypes and naive and memory IgM - arranged in rows), and the y-axis represents the Kyte-Doolittle values (the first column), Molecular Weight values (second column), Isoelectric Point values (third column) and the sum of the *V_H_* and *J_H_* gene lengths values (fourth column). The colors represent the fraction of clones with such a value. Blue colors are low frequencies, while red colors are high. The color bars near each plot represent significant and positive (red) or negative (blue) correlations between *M*(*V_H_,J_H_*) and the observed features. Green represents no significant correlations. The data were divided into 20 bins based on the chemical values distribution.

We computed a two-dimensional histogram for each measure for each isotype separately on the PREP dataset and (**Figure 5**). High *M*(*V_H_,J_H_*) values are associated with intermediate to low isoelectric points, molecular weights, and length, and a more complex picture for the KD. Specifically, *V_H_* and *J_H_* genes pair favor intermediate polarity and weight, but also some specific polarity of the resulting receptor. The correlations are strong in naive IgM and disappear for the switched B cells, supporting structural selection before the naive stage followed by antigen-specific selection (see Methods for statistical test).

## 4 Discussion

The human BCR repertoire is highly non-uniform, with preferred CDR3 length (35) and amino acid composition. Such preferences can be the result of the rearrangement process (36), antigen selection, or structural selection (9). Beyond CDR3 length distribution and composition, some *V_H_, J_H_* and *J_H_* segments are more frequent than others, *V_H_, D_H_, J_H_* usage is different among patients and conditions (37). Moreover, there is strong evidence that the *D_H_*-*J_H_* rearrangement is biased and some pairs are preferred (38), since *D_H_* and *J_H_* segments are located closely and joined together. However, *V_H_* and *J_H_* segments are physically separated and there is no direct event pairing the *V_H_* segment with *J_H_*. In fact, there is no reason to expect a correlation between them, unless mediated by joint preference for *D_H_* genes. We have shown a clear *V_H_J_H_* pairing, and found a large bias in the F clones but not in the NF clones. Moreover, the rearrangement-induced *V_H_, J_H_* pairing is not induced by their pairing to *D_H_* usage. We analyzed the evolution of deviation from random pairing in different peripheral compartments and found that the deviation from random pairing is most consistent in the naive IgM repertoire among samples, and then grows and diversifies in the memory and switched compartments. In parallel, the deviation from random pairing is strongly associated with multiple molecular properties of the receptors, including length (in AA), MW and polarity in the naive repertoire. The correlations decrease as the repertoire evolves to the switched memory compartments.

Our results suggest both positive and negative structural selection for pairing between *V_H_* and *J_H_*. This would be parallel to Linkage Disequilibrium (LD) in alleles. This deviation obviously does not imply that the *P*(*V_H_*)*P*(*J_H_*) is not a good predictor of *P*(*V_H_,J_H_*). Indeed, for most gene pairs, there is no significant deviation from random pairing. The most natural stage such a selection can occur is during positive and negative selection in the bone marrow. This is suggested by the bias in thenaive repertoire. BCRs that cannot bind at least weakly antigens or BCRS binding too strongly antigens in the bone marrow, for example, following excessive charge in the CDR3 may be selected against. Similarly, B cells carrying BCRs that can bind weakly many targets may be positively selected. This is consistent with the reported biased paratope usage in the naive repertoire (39).

Malfunctions of BCR repertoire development are associated with the pathogenesis of multiple immune-mediated diseases. Long CDR3 sequences in the BCR are associated with antibody polyreactivity and autoimmunity (40). Association between the length of CDR3 and the use of *V_H_* genes were found in healthy individuals (41, 42). Increased CDR3 length was found in SLE (IgG and IgA) and Crohn’s disease (unswitched B cells) (42). Furthermore, they showed that some individual genes and *V_H_* subgroups preferentially bind microbial antigens and/or have been associated with autoimmunity. The presented results show another aspect of the repertoire bias.

The current analysis was performed on healthy repertoires. We found no evidence that these pairs are directly associated with malfunctions. However, the reported shared patterns of *V_H_*-*J_H_* pairing can serve to detect deviations from this normal pattern that can be associated with malfunctions.

The evidence proposed here is indirect. We observe biases in the naive repertoire and propose a selection mechanism most consistent with the observations. Such a structural mechanism may be a crucial step in shaping the naive repertoire but is also important for the design of antibody libraries. While we have shown here one possible mechanism. Other selection mechanisms may exist that may be crucial for the design of such libraries.

## Data availability statement

The original contributions presented in the study are included in the article/**Supplementary Material**. Further inquiries can be directed to the corresponding author.

## Ethics statement

Ethical approval was not required for the study involving humans in accordance with the local legislation and institutional requirements. Written informed consent to participate in this study was not required from the participants or the participants’ legal guardians/next of kin in accordance with the national legislation and the institutional requirements.

## Author contributions

RL wrote the paper and performed all the analysis. YL supervised the analysis and wrote part of the paper. SD performed part of the analysis. All authors contributed to the article and approved the submitted version.
