# Supplementary material for: Shared bias in H chain V-J pairing in naive and memory B cells
Source: Front Immunol. 2023 Sep 18;14:1166116. doi: 10.3389/fimmu.2023.1166116 (PMC10543446; doi:10.3389/fimmu.2023.1166116)
Supplement: Supplementary file 1 [file DataSheet_1.pdf]

1 Supplementary Material

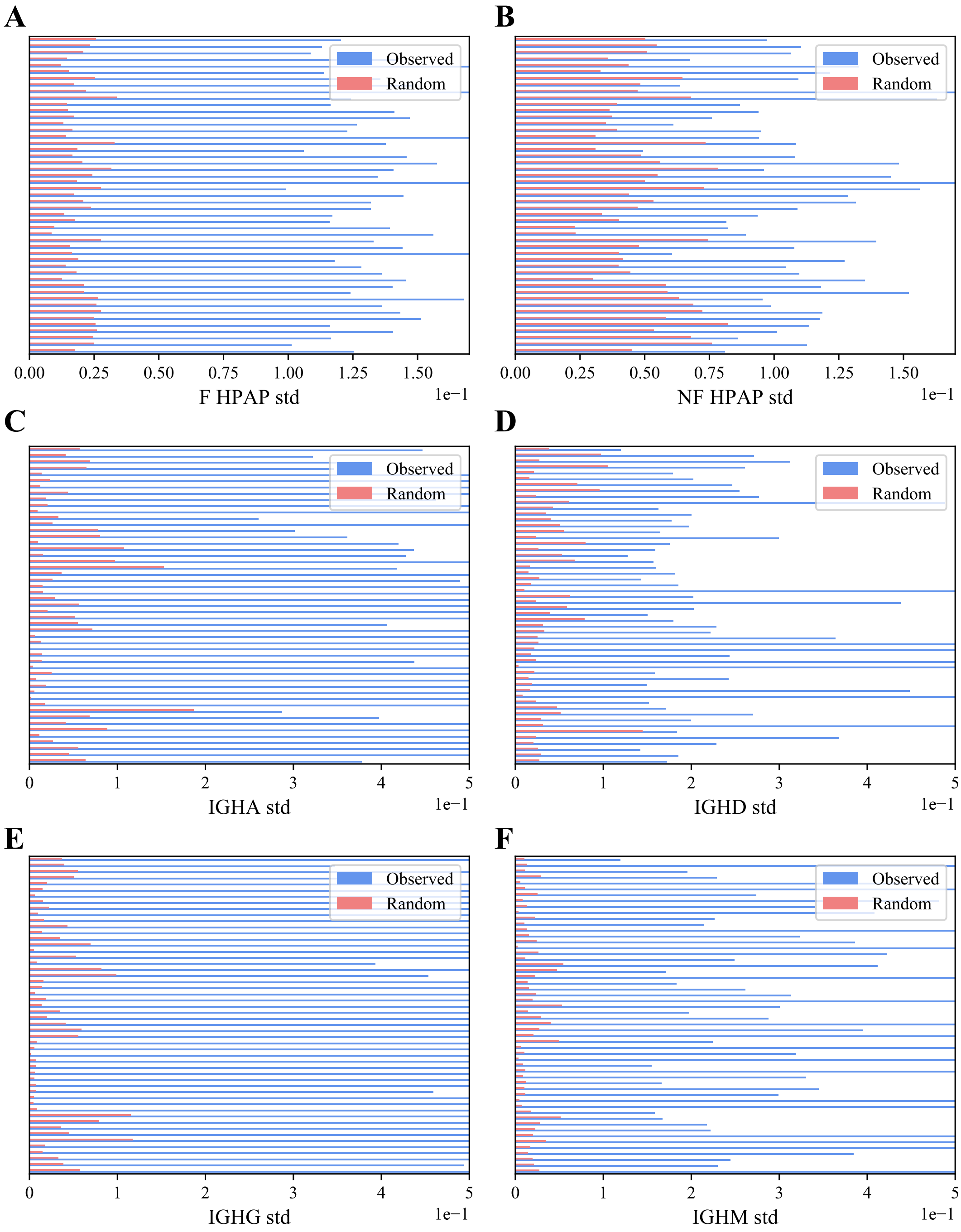

Figure S1: $M(V_H, J_H)$ bias with consideration of the frequency of each clone in each donor. The standard deviation of $M(V_H, J_H)$ values for the HPAP dataset (**A, B**) and the PREP dataset for each isotype separately (**C-F**). The blue bars describe the real F clones values (**A, C-F**) and the real NF clones values (**B**) while the pink bars represent the null model.
